# Durable tracking anti-SARS-CoV-2 antibodies in cancer patients recovered from COVID-19

**DOI:** 10.1038/s41598-021-96195-w

**Published:** 2021-08-30

**Authors:** Yongsheng Huang, Jing Yu, Dan Li, Kai He, Wenyang Liu, Lin Wang, Yeshan Chen, Conghua Xie, Xiaowei Wu

**Affiliations:** 1 School of Basic Medicine, Peking Union Medical College, Institute of Basic Medical Sciences, Chinese Academy of Medical Sciences, Beijing, China; 2 Department of Thoracic Surgery, TongJi Hospital, TongJi Medical College, Huazhong University of Science and Technology, Wuhan, 430000 China; 3 Department of Radiation and Medical Oncology, Zhongnan Hospital, Wuhan University, Wuhan, 430071 China; 4 Hubei Cancer Clinical Study Center, Zhongnan Hospital, Wuhan University, Wuhan, China; 5 Cancer Center, Union Hospital, Tongji Medical College, Huazhong University of Science and Technology, Wuhan, 430000 China; 6 National Cancer Center/Cancer Hospital, Peking Union Medical College, Chinese Academy of Medical Sciences, Beijing, China; 7 Guangdong Provincial Key Laboratory of Single Cell Technology and Application, School of Basic Medical Science, Southern Medical University, Guangzhou, China

**Keywords:** Infectious diseases, Cancer

## Abstract

Cancer patients are more susceptible to SARS-CoV-2 infection and generally have higher mortality rate. Anti-SARS-CoV-2 IgG is an important consideration for the patients in this COVID-19 pandemic. Recent researches suggested the rapid decay of anti-SARS-CoV-2 antibodies in the general population, but the decline rate of the antibodies in cancer patients was unknown. In this observational study, we reported the clinical features of the 53 cancer patients infected by SARS-CoV-2 from Wuhan, China and tracked the presence of anti-SARS-CoV-2 antibodies in the patients for more than 12 months. We found the duration (days) of anti-SARS-CoV-2 IgG in the patients was significant longer in chemotherapy (mean: 175; range: 75 to 315) and radiotherapy groups (mean: 168; range: 85 to 265) than in non-chemo- or radio-therapy group (mean: 58; range: 21 to 123) after their recovery from COVID-19. We also used single-cell RNA sequencing to track the immunologic changes in a representative patient recovered  from COVID-19 and found that CD8 + effective T cells, memory B cells and plasma cells were persistently activated in the patient undergoing chemotherapy. Together, our findings show that chemotherapy and radiotherapy might be beneficial to extend the duration of anti-SARS-CoV-2 IgG.

## Introduction

The emergence of coronavirus disease 2019 (COVID-19), caused by severe acute respiratory syndrome coronavirus 2 (SARS-CoV-2), has led to an unprecedented and ongoing global health crisis^1^. As of June 30th, 2021, World Health Organization (WHO) reported 19,718,030 confirmed COVID-19 cases in the world, including 3,937,437 deaths^2^. Cancer is also one of the most prevalent diseases worldwide^3^. In 2021, 1,898,160 new cancer cases and 608,570 cancer deaths are projected to occur in the United States^3^.

Cancer patients usually have weakened immune system and autoreactive responses^4^, and were reported to be more susceptible to SARS-CoV-2 infection and have higher mortality rate compared with regular COVID-19 patients^5,6^ . Therefore, cancer patients should be monitored more carefully during the treatment^5^, and the anti-SARS-CoV-2 antibodies are important as they improve the immunity of patients^7,8^ .Previous studies suggested the memory B cells (MBCs) against SARS-CoV-2 could be enriched for up for six months in the general convalescent patients^9^, while levels of anti-SARS-CoV-2 IgG antibody rapidly declined as early as three months after infection^10–12^. So far, to our knowledge, the duration of the antibodies in cancer patients has not been well established. In this study, we tracked the anti-SARS-CoV-2 antibodies in 53 cancer patients after their recovery from COVID-19 for more than 12 months, aiming to better comprehend the effects of different treatments on the durability of anti-SARS-CoV-2 antibodies and their impact on the immune system of COVID-19 cancer patients.

## Results

### Anti-SAS-Cov-2 IgG antibody has longer duration in the patients with chemotherapy and radiotherapy

**Figure 1:**
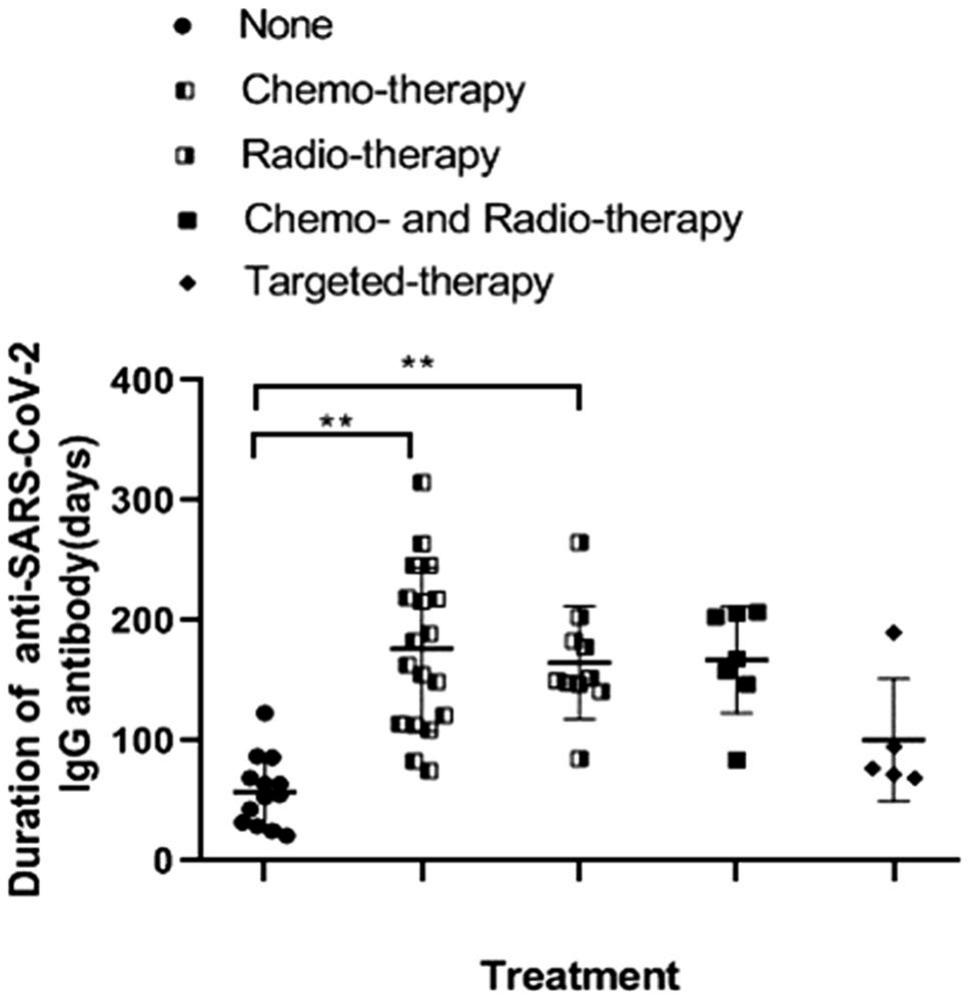
The duration of anti-SARS-CoV-2 IgG antibody in the cancer patients recovered from COVID-19 under different treatments. Kruskal–Wallis test followed by Mann–Whitney U test were used (*0.01 ≤ p < 0.05, **p < 0.01).

A total of 53 cancer patients (24 women and 19 men) who were infected by SARS-CoV-2 had serial measurements of IgG (Table 1). Infection was confirmed by polymerase chain-reaction assay in all participants. The mean age of patients was 56 years (range: 33 to 78). The mean duration of IgM is 28 days (range: 15 to 65), and the mean duration of IgG is 137 days (range: 21 to 315). There were 17 non-small-cell lung cancer (NSCLC), 6 breast cancer, 5 colon cancer, 5 cervical squamous cell carcinoma (CSCC) and 20 other types of cancer patients in this study (Supplementary Table 1). When we divided the participants into different groups by treatments, we found the duration of IgG was significantly longer in chemotherapy (mean: 175; range: 75 to 315; *p* < 0.01) and radiotherapy groups (mean: 168; range: 85 to 265; *p* < 0.01) than in non-chemo- or radio- therapy group (mean: 58; range: 21 to 123) (Fig. 1) (Table 1). The correlation between chemotherapy (Regression coefficient: 95.655; 95% confidence interval (CI): 35.702 ~ 155.608; *p* = 0.003) or radiotherapy (Regression coefficient:102.329; 95%CI: 38.107 ~ 166.551; *p* = 0.003) on antibody duration was further proved by a linear regression model (Table 2). However, we found that the duration of IgG was not significantly correlated with initial IgG levels, gender, cancer type, stage or underlying disease (Table 2).

**Table 1:** Clinical characteristics of the cancer patients recovered from COVID-19 under different treatments.

Variables	All	None	Chemotherapy	Radiotherapy	Cheo- and radio-therapy	Targeted therapy
Patient number	53	13	19	9	7	5
Male/female	29/24	6/7	10/9	5/4	5/2	3/2
Age (years)	56 (33–78)	58 (41–78)	53 (37–69)	61 (33–72)	57 (44–66)	55 (47–66)
Duration of IgM (days)	28 (15–65)	22 (16–28)	31 (21–51)	25 (15–30)	36 (23–65)	26 (15–33)
**Duration of IgG (days)**	137 (21–315)	58 (21–123)	175 (75–315)	168 (85–265)	168 (84–206)	101 (69–190)
≥ 90	34 (64.2%)	1 (7.6%)	17 (89.5%)	8 (88.9%)	6 (85.7%)	2 (40.0%)
≥ 180	8 (15.1%)	0 (0%)	9 (47.4%)	3 (33.3%)	3 (42.9%)	1 (25.0%)
≥ 240	6 (11.3%)	0 (0%)	4 (21.1%)	1 (11.1%)	1 (14.3%)	0 (0%)
**Initial blood features**						
IgG (ng/ml)	22.0 (1.8–80.4)	9.6 (5.9–17.2)	23.7 (6.9–56.4)	17.0 (6.4–52.6)	46.7 (12.4–80.4)	9.2 (1.8–15.4)
WBC (× 10^9^/L)	6.07 (1.56–19.30)	6.18 (2.77–13.12)	5.97 (1.56–10.58)	4.25 (3.67–7.61)	9.69 (4.04–19.30)	4.36 (3.02–4.90)
HB (g/dl)	115 (53–155)	115 (75–150)	118 (69–155)	107 (53–143)	101 (84–127)	132 (118–146)
PLT (× 10^9^/L)	172 (56–347)	222 (144–347)	160 (56–125)	146 (71–222)	156 (85–285)	136 (87–207)
LY (× 10^9^/L)	0.96 (0.21–2.19)	1.03 (0.29–1.41)	1.04 (0.35–2.01)	1.10 (0.34–2.19)	0.79 (0.27–1.97)	0.46 (0.21–0.66)
CRP (mg/L)	24.97 (0.16–196.3)	10.24 (2.30–80.44)	25.15 (0.16–196.3)	5.05 (0.79–13.72)	52.77 (1.11–126.9)	54.65 (1.63–175.7)

Data are numbers, mean (range) or n (%) unless otherwise indicated.

*WBC* white blood cell, *HB* haemoglobin, *PLT* platelet, *LY* lymphocyte, *CRP* C-reactive protein, *none* non-chemo or radio-therapy.

**Table 2:** Factors that affect the duration of anti-SARS-CoV-2 IgG antibody in cancer patients recovered from COVID-19 as analyzed with linear regression model.

Variables	Regression coefficient	95% CI	p value
Initial IgG levels	0.015	−1.303 to 1.417	0.933
**Cancer stage**			
Stage I	Ref	–	–
Stage II	14.860	−86.123 to 115.843	0.767
Stage III	13.489	−90.434 to 117.412	0.794
Stage IV	10.083	−92.691 to 112.856	0.843
Age	−1.816	−3.798 to 0.166	0.071
**Gender**			
Female	Ref	–	–
Male	11.625	−24.186 to 47.436	0.514
**Treatment**			
None	Ref	–	–
Chemotherapy	95.655	35.702 to 155.608	0.003*
Radiotherapy	102.329	38.107 to 166.551	0.003*
Chemotherapy + radiotherapy	95.186	10.611 to 179.760	0.028*
Target therapy	24.750	−46.835 to 96.334	0.488
**Underlying disease**			
No	Ref	–	–
Yes	8.770	−29.838 to 47.378	0.648

*p value less than 0.05 means statistically significant.

*CI* confidence interval.

### The immune system is continuously activated in the chemotherapy patient after the recovery of COVID-19

**Figure 2:**
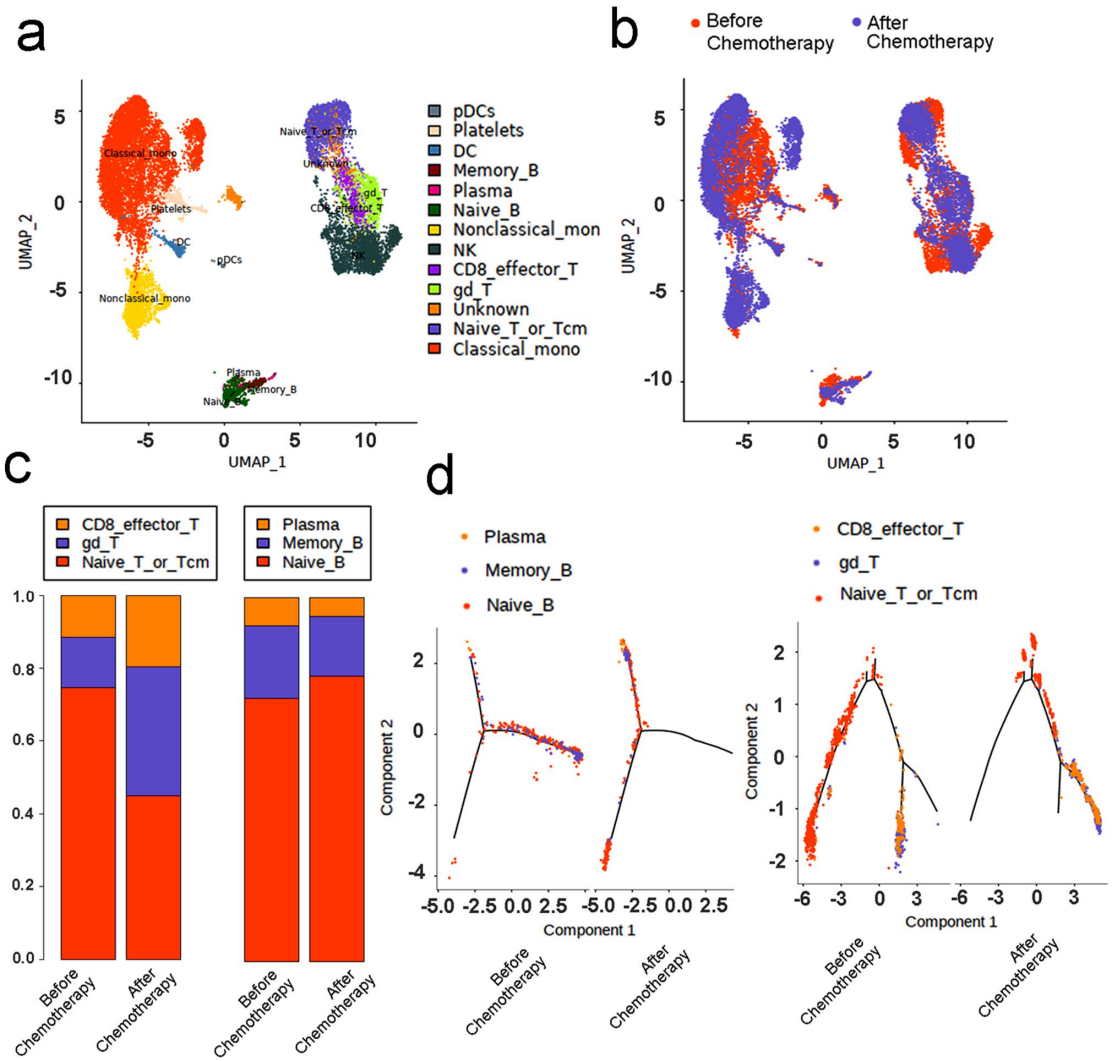
Dynamic study for the immunologic features in a representative patient infected by SARS-CoV-2 for more than 1 year. This 57-year-old male patient was diagnosed with non-small-cell lung cancer (stage: IIIA) and SARS-CoV-2 infection on February 25, 2020. After recovery from COVID-19, he underwent four chemotherapy cycles (500 mg/m^2^ pemetrexed combined with 75 mg/m^2^ Nedaplatin) from August to September 2020. The single-cell sequencing blood collections were conducted both before the chemotherapy (April 16, 2020) and after the chemotherapy (February 28, 2021). **(a)** UMAP projection of clusters by cell type (15, 015 cells), different colors corresponding to different cell types. **(b)** UMAP projection of clusters by sample source, red: before chemotherapy; blue: after chemotherapy (February 28, 2021). **(c)** Percentage of cell composition of each sample before and after chemotherapy; left: proportion of different T cell subset; right: proportion of different B cell subsets. **(d)** The Monocle 2 trajectory plot showing the dynamics of T cells (left) and B cells (right) in the patient before and after chemotherapy.

Interestingly, six participants (11.3%) in our cohorts showed durable presence of the anti-SARS-CoV-2 IgGs, which has already lasted for more 240 days (Table 1). Of them, four received chemotherapies, one received radiotherapy and one received both chemotherapy and radiotherapy after COVID-19 recovery. We collected peripheral blood mononuclear cells (PBMC) from one representative chemotherapy patient recoverd from COVID-19 and performed single-cell RNA sequencing. The uniform manifold approximation and projection (UMAP) (Fig. 2a–c) and trajectory analysis (Fig. 2d)showed the CD8 + effective T cells, memory B cells and plasma cells were persistently activated in this patient after chemotherapy.

## Discussion

It has been reported that SARS-CoV-2 could undergo evolution during the treatment of chronic infection^13–16^. Anti-SARS-CoV-2 IgG antibodies are important for the immunity of the cancer patients^7,8^. In this study, we found that the anti-SARS-CoV-2 IgG antibodies decayed fast in the patients without chemotherapy and radiotherapy, which is consistent with the previous finding in the general population^10–12^. However, our findings raise concern that human immunity against SARS-CoV-2 may be long lasting in patients with radiotherapy and chemotherapy. As we know, chemotherapy or radiotherapy can damage the immune system by destroying the hematopoietic stem cells in bone marrow, which may cause immunosuppression^7^. However, cell death caused by the chemotherapy or radiotherapy might also activate the adaptive immune system^17^, resulting in immunogenic cell death effect. Lee et al.^6^ found there was no significant effect on mortality for patients with chemotherapy and radiotherapy use within the 4 weeks after testing positive for COVID-19. Hess et al.^18^ reported low-dose, whole-lung radiation for patients with COVID-19-related pneumonia appeared safe and might be an effective immunomodulatory treatment. Besides, our group^19^ and one group in Italy^20^ showed that very few patients required treatment interruptions in radiotherapy services, and few patients undergoing radiotherapy were diagnosed with COVID-19 during their treatment course (0.48%, 1 of 209 patients)^19^. Thus, chemotherapy and radiotherapy should be safe treatments for the cancer patient recovered from COVID-19. Interestingly, there is also a report that anti-SARS-CoV-2 antibody triggered the anti-tumor immune response in a Hodgkin's lymphoma patient^21^. Therefore, the protective role of IgG antibodies against SARS-CoV-2 in the cancer patient is not only important for them to prevent virus infection, but maybe also beneficial for the cancer treatment.

Our study has several limitations. Firstly, this is a multicentric study which performed mainly in two hospitals in Wuhan. We have used different commercial assay Kits to detect anti-SARS-CoV-2 IgG and we could not acquire all the information such as IgG expression levels of patients at each time point. Thus, we mainly focused on the duration but not the expression level of IgG antibody. Secondly, some cancer patients were discharged, died or in unstable physical condition in the process, which resulted in a relatively small sample size. Thirdly, some patients have been used supportive treatments to maintain a normal white blood cell count or hemoglobin level, maintain electrolyte balance and ensure adequate intake. The uncertainties of these different supportive treatments might also affect the duration of anti-SARS-CoV-2 IgG.

In sum, to the best of our knowledge, this study first report that chemotherapy and radiotherapy might provide benefits to prolong the duration of anti-SARS-CoV-2 IgG in human body. This should be important to devise new strategies for cancer treatment and improve antibody therapy in the future. Still, further large-scale investigations on IgG antibodies against SARS-CoV-2 in cancer patients over longer time periods should be done to assess the kinetics of immunity.

## Methods

### Patient data

We reviewed the medical records, including clinical and treatment data of patients with cancer who were mainly admitted to the Zhongnan Hospital of Wuhan University and Wuhan Tongji Hospital from February 1, 2020, to March 31, 2021. COVID-19 infection was confirmed by polymerase-chain-reaction (PCR) assay. The chat flow of the cancer patients in the study was shown in Supplementary Fig. 1, and the detailed information and clinical features of patients were shown in Supplementary Table 1. During the treatment of patients, venous blood samples were serially collected and analyzed by gold immunochromatography assay (GICA) or enzyme-linked immunosorbent assay (ELISA) or to detect anti-SARS-CoV-2 IgG/IgM. In Tongji hospital, we used one ELISA Kit (EknCov-S1-01, Frdbio bioscience and technology Inc.) and one gold immunochromatography assay (GICA) Kit (200101, Wuhan Easydiagnosis biomedicine Co.Ltd.). In Zhongnan hospital, we mainly used another GICA Kit (20203400240, Zhuhai Livzon Diagnositic Inc.). For the reaction of ELISA^22^, optical density at 450 nm (OD450) was determined with a multifunctional microplate reader. The cutoff for IgG was 0.30 determined by calculating the mean OD450 of a negative serum sample plus 3 SDs. Duration of SARS-CoV-2 antibody among the patients were recorded. All the patients selected in this study were alive before the cutoff date (March 31, 2021), and verbal informed consent was obtained from all the participants. All methods were carried out in accordance with relevant guidelines and regulations.

### Single-cell RNA sequencing

Peripheral blood mononuclear cells (PBMCs) were collected from one representative chemotherapy patient using a Ficoll–Hypaque density solution according to the standard density gradient centrifugation methods. This 57-year-old male patient was diagnosed with non-small-cell lung cancer (stage: IIIA) and SARS-CoV-2 infection on February 25, 2020. After recovered from COVID-19, the first blood collection for single-cell RNA sequencing (Singeron) was conducted on April 16, 2020. The patient underwent four chemotherapy cycles (500 mg/m^2^ pemetrexed combined with 75 mg/m^2^ Nedaplatin) from August to September 2020. The second blood collection for single-cell RNA sequencing was conducted on February 28, 2021. After quality control, we used Seurat v3.8 to do data normalization, dimensional reduction, clustering and calculated differentially express genes (DEGs) among clusters. We identified cell types (15,015 cells) base on DEGs and CellMarker database.

### Statistics

Statistical analysis in Fig. 1 was performed using Prism 7 software (GraphPad La Jolla, USA). Kruskal–Wallis test followed by Mann–Whitney U test were used, p value less than 0.05 was considered to be statistically significant. Linear regression model in Table 2 was performed by the lme4 and lmerTest packages in R version 3.6.1, p value less than 0.05 means statistically significant.

### Study approval

This retrospective study was approved by the ethics committee of Wuhan Tongji Hospital (2020370) and Zhongnan Hospital of Wuhan University (2020039).

## Supplementary Information

Supplementary Information.

## Data Availability

All data generated or analysed during this study are included in this published article (and its Supplementary Information files).

## Abbreviations

COVID-19: Coronavirus disease 2019
SARS-CoV-2: Severe acute respiratory syndrome coronavirus 2
WHO: World Health Organization
MBCs: Memory B cells
PBMC: Peripheral blood mononuclear cell
NSCLC: Non-small-cell lung cancer
CSCC: Cervical squamous cell carcinoma
GICA: Gold immunochromatography assay
ELISA: Enzyme-linked immunosorbent assay
