# Supplementary material for: Durable tracking anti-SARS-CoV-2 antibodies in cancer patients recovered from COVID-19
Source: Sci Rep. 2021 Aug 30;11:17381. doi: 10.1038/s41598-021-96195-w (PMC8405618; doi:10.1038/s41598-021-96195-w)
Supplement: Supplementary file 1 — Supplementary Information. [file 41598_2021_96195_MOESM1_ESM.pdf]

Supplementary figure 1: Flow diagram of cancer patients with SARS-CoV-2 infection

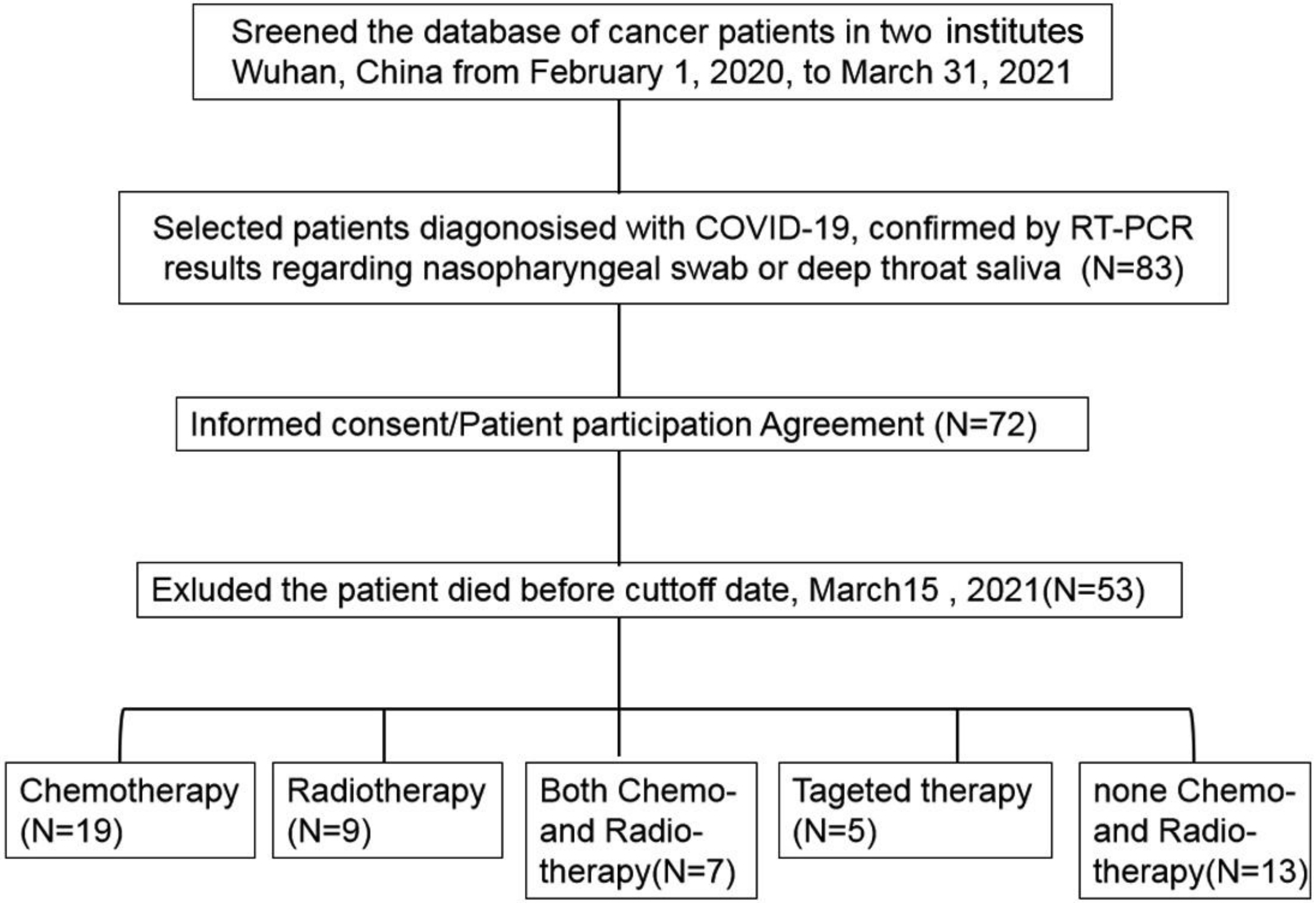

Supplementary table 1. Clinical characteristics and treatments of the cancer patients after COVID-19 diagnosis

				Duration of Antibody							First test of blood risk factors after diagnosis				
Patient	Gender	Age	Diagnosed date	IgG	IgM	Treatment	Cancer type	Stage	Underlying Disease	Initial IgG	WBC(G/L)	HB(G/L)	PLT(G/L)	LY(G/L)	CRP(G/L)
1	Female	51	2020/3/22	123	28	None	CSCC	II	None	9.1	3.44	114	180	1.39	/
2	Female	33	2020/4/8	>265	27	Radiotherapy	CSCC	III	None	16.8	3.67	53	222	1.19	/
3	Female	61	2020/4/28	86	21	None	CSCC	I	Hypertension	/	2.77	123	144	0.74	2.3
4	Female	56	2020/4/30	69	19	None	CSCC	III	None	17.2	12.7	80	347	1.24	80.44
5	Female	56	2020/5/8	113	21	Chemotherapy	CSCC	III	None	6.9	6.86	102	246	1.21	9.69
6	Female	63	2020/3/24	83	24	Chemotherapy	Ovarian	IV	Diabete	10.8	8.51	108	56	2.01	69.85
7	Female	56	2020/4/15	216	18	Chemotherapy	Ovarian	II	None	28	8.31	132	138	1.1	0.16
8	Female	41	2020/4/17	>246	33	Chemotherapy	Ovarian	III	Hypertension	23.5	6.2	141	227	1.6	14.84
9	Female	66	2020/3/29	147	28	Chemotherapy+Radiotherapy	Endometrial	III	None	12.4	6.48	127	285	1.97	1.11
10	Female	35	2020/3/22	183	29	Chemotherapy	Vestibular gland	II	None	19.9	6.47	125	261	0.9	/
11	Female	45	2020/2/29	75	21	Chemotherapy	Breast	III	None	53.7	5.12	78	216	0.7	1
12	Female	52	2020/3/15	203	37	Chemotherapy+Radiotherapy	Breast	III	None	46.3	4.51	96	145	1.03	17.4
13	Female	49	2020/4/16	87	25	None	Breast	II	None	9.1	5.74	113	292	1.39	2.97
14	Female	67	2020/4/30	152	21	Radiotherapy	Breast	II	Hypertension	17.7	4.33	104	115	0.72	8.9
15	Female	43	2020/5/6	189	27	Chemotherapy	Breast	III	None	43.5	5.6	155	215	0.54	4
16	Female	60	2020/5/23	178	37	Radiotherapy	Breast	II	None	52.6	1.75	121	108	0.63	1.1
17	Male	57	2020/4/9	25	16	None	Colon	II	None	/	6.85	75	199	1.02	4.1
18	Female	60	2020/4/11	121	39	Chemotherapy	Colon	IV	Heart disease	26.8	4.09	118	192	1.27	2.3

19	Male	54	2020/4/28	>219	28	Chemotherapy	Colon	III	None	8.7	4.96	69	123	0.85	0.74
20	Male	62	2020/4/30	114	28	Chemotherapy	Colon	III	None	16.3	4.26	127	156	0.54	7.15
21	Male	47	2020/5/29	95	31	Target therapy	Colon	II	None	10.5	3.02	142	207	0.66	1.63
22	Male	66	2020/3/4	72	15	Target therapy	Small intestine	II	None	1.8	3.54	146	127	0.21	20.03
23	Male	63	2020/4/28	21	18	None	Rectal	II	Diabete	/	13.12	125	202	0.77	3.28
24	Male	37	2020/3/9	218	24	Chemotherapy	Hepatocellular	II	Viral hepatitis B	35.1	7.45	138	155	1.54	2.3
25	Female	63	2020/5/15	29	24	None	Hepatocellular	III	Hypertension	5.9	7.79	142	227	1.16	5.81
26	Male	58	2020/2/1	64	23	None	Gastric	II	None	9.4	4.25	/	/	1.37	3.9
27	Male	69	2020/3/3	149	28	Chemotherapy	Gastric	III	None	12.5	3.84	/	/	1.98	7.3
28	Male	51	2020/3/18	109	37	Chemotherapy	Gastric	III	Diabete	12.6	1.56	132	128	0.51	1.6
29	Male	51	2020/4/4	64	22	None	Gastric	III	None	/	4.95	128	186	0.87	3.68
30	Male	37	2020/2/29	>246	36	Chemotherapy	Non Hodgkin's lymphoma	IV	Pulmonary tuberculosis	56.4	5.2	107	90	1.29	0.64
31	Male	78	2020/3/8	43	21	None	Vertebral	I	Diabete	14	5.86	126	173	1.13	4.41
32	Female	64	2020/5/8	147	26	Radiotherapy	Nasopharyngeal	III	None	15.9	5.78	/	/	1.89	/
33	Male	61	2020/5/15	203	26	Radiotherapy	Nasopharyngeal	III	None	6.8	3.47	/	/	1.24	0.79
34	Female	41	2020/5/7	55	28	None	PCC	I	None	/	4.24	104	168	0.29	5.39
35	Male	58	2020/4/29	163	34	Chemotherapy	SCLC	III	None	23.6	2.5	133	188	0.99	0.46
36	Male	61	2020/5/7	158	28	Chemotherapy+Radiotherapy	SCLC	IV	Heart disease	41.5	14.77	/	/	0.36	126.9
37	Male	57	2020/2/25	>315	25	Chemotherapy	NSCLC	III	None	33.3	3.92	130	89	0.44	14.3
38	Male	61	2020/3/26	150	22	Radiotherapy	NSCLC	II	Kidney insufficiency	6.4	4.18	78	147	0.34	13.72
39	Male	56	2020/3/30	84	65	Chemotherapy+Radiotherapy	NSCLC	IV	None	54.8	19.3	/	/	0.82	84.9
40	Female	48	2020/4/7	69	29	Target therapy	NSCLC	II	Heart disease	8.5	6.67	122	87	0.46	20.85
41	Male	63	2020/4/11	85	23	Radiotherapy	NSCLC	III	None	17.1	7.61	114	176	2.19	6.2
42	Male	55	2020/4/18	53	19	None	NSCLC	I	None	7.6	3.58	150	289	0.62	2.8

43	Male	64	2020/4/21	264	51	Chemotherapy	NSCLC	IV	Pulmonaryem physema	8.5	8.52	/	/	0.35	196.3
44	Male	65	2020/4/27	168	23	Chemotherapy+Radiotherapy	NSCLC	IV	None	80.4	4.04	84	108	0.66	4.45
45	Male	58	2020/4/30	155	46	Chemotherapy	NSCLC	IV	None	14.1	9.46	/	/	0.72	115.3
46	Female	66	2020/5/18	32	26	None	NSCLC	IV	Hypertension	6.5	4.99	98	259	1.41	3.77
47	Male	49	2020/5/20	77	21	Target therapy	NSCLC	IV	None	15.4	3.65	118	124	0.57	/
48	Male	72	2020/5/21	183	15	Radiotherapy	NSCLC	II	Hypertension	11.3	3.92	143	71	0.9	2.21
49	Male	70	2020/5/24	148	30	Radiotherapy	NSCLC	II	None	8.4	3.57	133	186	0.78	2.44
50	Male	55	2020/5/29	207	31	Chemotherapy+Radiotherapy	NSCLC	III	None	25.6	8.39	95	85	0.27	16.54
51	Male	44	2020/6/19	>206	43	Chemotherapy+Radiotherapy	NSCLC	IV	None	65.6	10.34	/	/	0.4	118.1
52	Female	66	2020/7/5	190	33	Target therapy	NSCLC	IV	Hypertension	9.8	4.9	/	/	0.38	175.7
53	Female	63	2020/7/15	141	38	Chemotherapy	NSCLC	IV	None	16.3	10.58	98	72	1.18	4.8

Abbreviations: CSCC,cervical squamous cell carcinoma; PCC,pheochromocytoma; NSCLC,non small-cell lung cancer; SCLS,small cell lung cancer.
